# Integrated population pharmacokinetics of etirinotecan pegol and its four metabolites in cancer patients with solid tumors

**DOI:** 10.1007/s00280-018-3562-3

**Published:** 2018-03-21

**Authors:** Sherwin K. B. Sy, Yen Lin Chia, Toufigh Gordi, Ute Hoch, Michael A. Eldon

**Affiliations:** Department of Clinical Pharmacology, Nektar Therapeutics, 455 Mission Bay Blvd South, San Francisco, CA 94158 USA

**Keywords:** Etirinotecan pegol, Irinotecan, SN38, Breast cancer, Population pharmacokinetics

## Abstract

**Purpose:**

Etirinotecan pegol (EP), a long-acting topoisomerase-1 inhibitor, is a polyethylene glycol conjugate of irinotecan, with an intended indication for treatment of breast cancer with brain metastases. The objective of this study was to develop a population pharmacokinetic (popPK) model of EP and four of its metabolites (irinotecan, SN38, SN38-glucuronide, and APC) and determine covariates affecting their pharmacokinetics.

**Methods:**

Data from 83 cancer patients enrolled in phase 1 studies were used. The model was developed in two stages: (1) concentration–time data were analyzed with a 3-analyte model for EP, irinotecan, and SN38; and (2) a 5-analyte model developed based on expansion of 3-analyte model to include concentration–time data for SN38 glucuronide and APC with parameter values from 3-analyte model fixed. Covariate relationships with parameters were selected based on Wald’s test within the Wald’s Approximation Method approach, first for the 3-analyte model then the 5-analyte model.

**Results:**

The final integrated popPK model for the five analytes was a two-compartment per analyte model that followed the metabolic cascade of EP to irinotecan, followed by metabolism of irinotecan to the previously known metabolites, but with altered exposures as compared to administration of irinotecan. With the model developed based on total dose of EP, the population estimates of EP clearance and central volume were 0.237 L/h and 5.5 L, respectively. Patient age, body surface area (BSA), and estimated glomerular filtration rate were found to correlate with EP clearance and BSA with EP central volume. Individuals who were homozygous for UGT1A1*28 genotype had modestly reduced elimination capacity of SN38 compared to heterozygous and wild-type genotypes. Simulations evaluating the clinical importance of significant covariates indicated minimal change in areas under the curve and peak concentrations of EP and SN38.

**Conclusions:**

The pharmacokinetics of EP and four metabolites including the active metabolite SN38 were described by an integrated popPK model. Other than BSA, which was already accounted by a BSA-based dosing scheme, no other covariates were deemed to have clinical implications. No EP starting dose adjustment based on patient demographics and other covariates was deemed necessary.

**Electronic supplementary material:**

The online version of this article (10.1007/s00280-018-3562-3) contains supplementary material, which is available to authorized users.

## Introduction

Irinotecan, a prodrug of 7-ethyl 10-hydroxycamptothecin (SN38), is an antineoplastic agent of the topoisomerase (Top1) inhibitor class that is widely used to treat colorectal and other gastrointestinal cancers. The pharmacokinetics and metabolism of irinotecan and SN38 in humans are well described in the literature [1–5]. Irinotecan is extensively metabolized in the liver to various metabolites. It is cleaved enzymatically by carboxylesterases to form SN38, which has cytotoxic activity that is 100- to 1000-times greater than that of the parent drug [6]. SN38 is subsequently conjugated to an inactive glucuronide (SN38G) by uridine diphosphate glucuronosyltransferases (UGT1A1 and UGT1A9). Other inactive irinotecan metabolites are 7-ethyl-10-[4-*N*-(5-aminopentanoic acid)-1-piperidino]-carbonyloxycampthothecin (APC) and 7-ethyl-10-[4-amino-1-piperidino]-carbonyloxycamptothecin (NPC), resulting from metabolism of irinotecan by CYP3A4 and 3A5 enzymes [3, 7, 8].

**Fig. 1:**
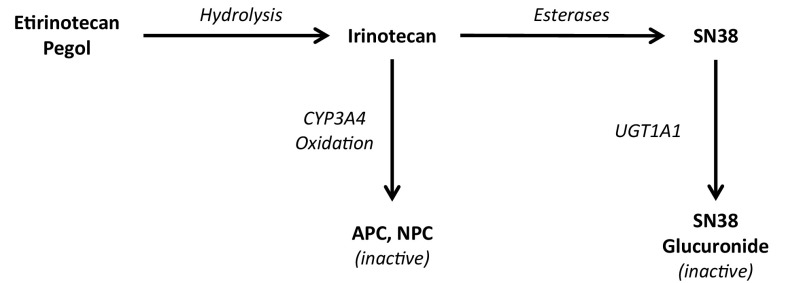
Etirinotecan pegol metabolic pathway

Etirinotecan pegol (EP) is a polyethylene glycol (PEG) conjugate of irinotecan, with an intended indication for treatment of breast cancer with brain metastases (BCBM). EP was designed to provide enhanced anti-tumor efficacy and a favorable tolerability profile through a modulated PK profile that facilitates lower peak plasma levels and sustained exposure of tumor tissue to SN38. In animal models, EP provided increased anti-tumor activity and a better safety profile compared with short-acting Top1 inhibitors [9, 10]. EP is a prodrug of irinotecan, consisting of a 20 kDa 4-arm PEG with a single irinotecan molecule conjugated to each arm via a glycine ester; the metabolic pathway of EP is shown in Fig. 1. Chemical hydrolysis of the glycine ester releases irinotecan in vivo; once released, irinotecan is metabolized to the previously described metabolites. The large molecular weight PEG of EP combined with the slow release of irinotecan results in 5–10-fold lower maximal plasma SN38 concentrations and a greatly prolonged half-life compared to that resulting from administration of irinotecan (approximately 40 days compared to about 2 days) [1, 11]. A single intravenous (IV) dose of 145 mg/m^2^ EP results in approximately the same plasma SN38 AUC as a 350 mg/m^2^ dose of irinotecan, but exposure is continuous throughout the 21-day cycle, rather than intermittent, and maximal concentrations are less after EP administration than after irinotecan administration [11].

This current analysis aimed to establish a semi-mechanistic population pharmacokinetic model that describes the metabolic pathway of EP following intravenous administration of single and multiple EP doses in patients with advanced cancer.

## Methods

### Clinical studies and patients

Data from two clinical trials of EP, 06-IN-IR001 and 07-PIR-02, in patients with advanced solid tumors were combined for popPK analysis. Both trials were approved by the Institutional Review Board at the study sites and were in compliance with the Declaration of Helsinki; all patients provided written informed consent. Study 06-IN-IR001 was a multicenter, open-label, phase 1 dose-escalation study for finding the maximum tolerated dose, as described previously [11]. Briefly, patients received EP by intravenous infusion over a 90-min period with 3–6 patients per dose cohort in the following regimens: (1) 3 once-weekly doses repeated every 4 weeks (wx3 q4wk); (2) once-biweekly (q14d); and (3) once every 3 weeks (q21d). Patients continued to the next cycle if they did not exhibit unacceptable toxicity or disease progression. Study 06-IN-IR001 enrolled a total of 76 patients; pharmacokinetic data from 67 patients were included for popPK modeling. 07-PIR-02 was the phase 1 portion of a multicenter, open-label, phase 2 safety, and efficacy study of EP in combination with cetuximab in patients with solid tumors refractory to the standard therapies [12]. Eighteen patients received EP (100 or 125 mg/m^2^) once every 3 weeks. Standard cetuximab schedule was administered as described [13]. Pharmacokinetic data from 16 patients were included in the popPK modeling. Reasons for exclusion of patients from the pharmacokinetic analysis are provided in the supplemental material. PK samples were collected at the beginning of day 1 and up to 4 weeks after the last dose. Rich blood PK sampling was carried out after the first and third doses in 06-IN-IR001 and after the first dose in 07-PIR-02, followed by sparse predose only sampling thereafter for the duration of treatment. All patients were evaluated for UGT1A1*28 genotype.

### Bioanalytical assays

Plasma EP and metabolite concentrations were determined using specific and validated LC–MS/MS methods previously described [14]. Prior to popPK analysis, etirinotecan pegol concentrations were converted, such that they reflected the irinotecan content using irinotecan loading factors of 9.4% (06-IN-IR001) and 9.5% (07-PIR-02). All analyte concentrations were converted to molar concentrations based on irinotecan, SN38, SN38G, and APC molecular weights of 586.678, 382.404, 568.53, and 618.687 amu, respectively. Samples below the limit of quantification (BLQ) were excluded and treated as missing in the analysis. The percentage of BLQ samples for EP, irinotecan, SN38, SN38G, and APC were 22.6, 3.8, 4.1, 6.1, and 36.4%, respectively.

### Pharmacokinetic model structure

Individual plasma concentration–time data were analyzed by non-linear mixed-effects modeling using stochastic approximation expectation maximization followed by importance sampling methods in Monolix 2016 (Lixoft, Antony, France). Graphical and statistical evaluations used during model development were generated in R 3.0.0 (R Foundation for Statistical Computing, Vienna, Austria) or Monolix. Model evaluation was based on the likelihood objective function value (OFV) result from the importance sampling procedure, Schwartz Bayesian Criterion (SBC), goodness-of-fit plots, and visual predictive check. Corrections for prediction and variability were incorporated in the visual predictive check [15–17].

Interindividual variability and residual error models were incorporated in the model, assuming log-normal distribution and a mixture of additive and proportional error models, respectively. A zero-centered mean was also assumed for the variability models.

### Covariate screening

The covariates considered in the model are based on the information in irinotecan product labeling, as well as the literature on its pharmacokinetics and metabolism, and are summarized in Table 1. Hepatic and renal functions, age, gender, UGT1A1 status, smoking, race, and concomitant administration of CYP inhibitors and inducers were investigated for their effects on the disposition of EP and metabolites.

**Table 1:** Potential covariates and rationales for their evaluation for effects on EP and metabolite disposition

Covariate name; variable type	Clinical importance	Information from irinotecan prescribing information	Comments	Disposition
ALT (U/L); continuous	Indicator of hepatic function	Irinotecan CL ↓ and SN38 exposure ↑ in hepatic impairment; effect proportional to degree of impairment ↑ Gr 3–4 neutropenia in pts with Bili > 1; DO NOT Dose if > 2	Correlated with bilirubin	Removed; bilirubin used as indicator of hepatic function
AST (U/L); continuous	Indicator of hepatic function		Correlated with ALT	Removed; bilirubin used as indicator of hepatic function
Bilirubin (mg/dL); continuous	Indicator of hepatic function		Most sensitive indicator for irinotecan in patients with hepatic impairment	Retained as indicator of hepatic function
eGFR CG (mL/min); continuous	Indicator of renal function	Not investigated in renal impairment-use with caution	Calculated from serum creatinine using Corrected CKD-EPI	Retained as indicator of renal function
Age (year); continuous	Potential for ↓CL with ↑ age	~ 11% ↑in SN38 AUC @ Age > 65 Reduce starting dose for q21d if age > 70 (300 mg/m^2^ vs. 350)		Retained as potential clinical covariate
BSA (m^2^); continuous	Indicator of body size			Retained as indicator of body size
Gender (F, M); categorical	Known clinical covariate for many drugs	No apparent difference		Retained as potential clinical covariate
UGT genotype; categorical	Potential indicator of SN38 CL	Homozygous pts at ↑ risk of neutropenia		Retained as indicator of UGT homozygosity
Smoking; categorical	Induction or Inhibition of metabolic CL	Not investigated for irinotecan		Retained as potential clinical covariate
Race; categorical	Known clinical covariate for many drugs	Influence of race not investigated for irinotecan		Enrollment of very few non-Caucasian patients precluded meaningful analysis

Baseline values for body weight, gender, age, creatinine, total bilirubin, AST, ALT, and albumin were used. Estimated GFR (eGFR) was calculated using the following equation [18]:

1 $${\text{GFR}}\;{\text{(mL/min/1.73}}\,{{\text{m}}^2}{\text{)}}=175 \cdot S_{{{\text{Cr}}}}^{{ - 1.154}} \cdot {\text{Ag}}{{\text{e}}^{ - 0.203}} \cdot {(0.742)^{I({\text{Female}})}} \cdot {(1.212)^{I({\text{Black}})}},$$

where *S*_cr_ is serum creatinine in mg/dL [19]. UGT1A1 status was determined based on the presence of the UGT1A1*28 [number of TA(7)] repeats in the promoter region of the UGT1A1 allele in whole blood samples using the FDA-approved Invader^®^ UGT1A1 Molecular Assay kit (Third Wave Technologies Inc., Madison, WC, USA). Results were reported as UGT1A1*28 not detected (wild-type or TA6/TA6), one copy detected (heterozygous or TA6/TA7), two copies detected (homozygous or TA7/TA7), or indeterminate (rare genotype with either TA5 or TA8). Patients were categorized as wild type, heterozygous for UGT1A1*28, or homozygous for UGT1A1*28 (Table 2). Patients reported as indeterminate were placed into the wild-type category.

Visual inspection using plots of covariate vs. standardized random effects of the parameters were utilized during the covariate screening process. Covariates that exhibited trends in the plots of standardized random effects were further screened using the Wald Approximation Method (WAM) developed by Kowalski and Hutmacher [20]. The following covariate relationships were formally evaluated with the WAM algorithm: BSA (vs. CL, *V*_1_), gender (vs. ${k_{3{\text{e}}}}$), age (vs. CL), eGFR (vs. CL), and UGT1A1 (vs. ${k_{3{\text{e}}}}$). The ten covariate models with the largest decrease in the SBC determined from the WAM algorithm were then incorporated to the popPK model to evaluate for their relationship to specific model parameters. A covariate was considered significant if the *p* value of the Wald test was ≤ 0.01.

The continuous covariates were incorporated as a power model, such that ${\theta _i}={\theta _{{\text{pop}}}}\prod\nolimits_{j} {{{\left( {\frac{{{\text{co}}{{\text{v}}_{i,j}}}}{{{\text{co}}{{\text{v}}_{{\text{median}},j}}}}} \right)}^{{k_{{\text{cov}},j}}}} \cdot \exp ({\eta _\theta }),}$ whereas categorical covariates were incorporated as an exponential model: ${\theta _i}={\theta _{{\text{pop}}}}\prod\nolimits_{j} {{\text{exp(}}{k_{{\text{cov}},j}}I[{\text{co}}{{\text{v}}_{i,j}}]{\text{)}} \cdot \exp ({\eta _\theta }),}$ with *i* being an index for individual with a specific covariate characteristic, *j* being the index for the covariate, pop for the index for the population typical value, *θ* representing the PK model parameter, cov is either a categorical or continuous covariate, *k*_cov_ is the coefficient that characterizes the covariate effect on the parameter, and *η*_*θ*_ refers to between-subject variability.

The potential clinical impact of statistically significant covariates on analyte exposure was evaluated by comparing the area under the concentration–time curve (AUC) and peak drug concentration (*C*_max_) values from 500 simulated profiles from virtual patients receiving six cycles of 145 mg/m^2^ once every 3 weeks. These virtual patients had differing categories of renal function, age (45 and 75 years), and UGT1A1*28 genotype. Simulated parameter values for each candidate covariate were compared to a reference population simulated using median covariate values using Forest plots.

## Results

### Patient characteristics and pharmacokinetic data set

The data set included data from 83 patients, 67 from study 06-IN-IR001, and 16 from study 07-PIR-02. Baseline characteristics of the patient population in the analysis data set are shown in Table 2. The majority of patients in the PK population were white with a median age of 60 years; 54% were male. Patients had a variety of cancer types, including colorectal (22.9%), lung (18.1%), and pancreatic (10.8%) cancers. Patients were required to have adequate renal function to be eligible to participate in both studies. Nonetheless, several patients exhibited mild and moderate renal impairment based on eGFR, allowing the assessment of the impact of renal function on EP pharmacokinetics. As expected from a mainly Caucasian patient population, 10.8% of patients were homozygous for UGT1A1*28. Fifteen percent and 45% of patients received concomitant weak/moderate CYP3A4 inhibitors and inducers, respectively.

**Table 2:** Characteristics of patients whose data were included in the development of the population pharmacokinetic model

Characteristics, (*N* = 83)	Median [range] or % of patients (*n*)
Patient demographics	
Weight (kg)	72.3 [43.9, 153.6]
Age (years)	60 [25, 81]
Body surface area (m^2^)	1.86 [1.36, 2.74]
Gender	
Male (%)	54.2% (45)
Female (%)	45.8% (38)
Ethnicity	
White (%)	94% (78)
Others (%)	6% (5)
Laboratory measurements	
Albumin (g/L)	36 [14, 45]
ALT (U/L)	21 [7, 153]
AST (U/L)	24 [11, 130]
Bilirubin (µmol/L)	10.3 [3.4,27.4]
Creatinine (µmol/L)	79.6 [35.4,132.6]
eGFR (mL/min/1.73 m^2^)	79.1 [34.9,216.6]
Cancer type	
Colorectal (%)	22.9% (19)
Lung (%)	18.1% (15)
Pancreas (%)	10.8% (9)
Breast (%)	3.6% (3)
Ovarian (%)	6% (5)
Others^a^ (%)	38.6% (32)
Number of UGT1A1*28 alleles present	
None (%)	38.6% (32)
One (%)	48.2% (40)
Two (%)	10.8% (9)
Indeterminate	2.4% (2)
Concomitant medications of CYP3A4 inhibitors/inducers	
Inhibitors	
Weak/moderate (%)	14.5% (12)
Strong (%)	–
Inducers	
Weak/moderate (%)	44.6% (37)
Strong (%)	–
Renal function category, eGFR	
Normal ≥ 90 mL/min	31% (26)
Mild impairment 60–89 mL/min	48% (40)
Moderate impairment 30–59 mL/min	19% (16)

^a^Cancer types that were < 10% in both studies, except for breast and ovarian cancer, which are listed separately

The pharmacokinetic data set consisted of 1414, 1777, 1769, 1731, and 1167 concentration values for model building for EP, irinotecan, SN38, SN38G, and APC, respectively.

### Etirinotecan pegol pharmacokinetics and covariate selection

#### 3-Analyte pharmacokinetic model

**Fig. 2:**
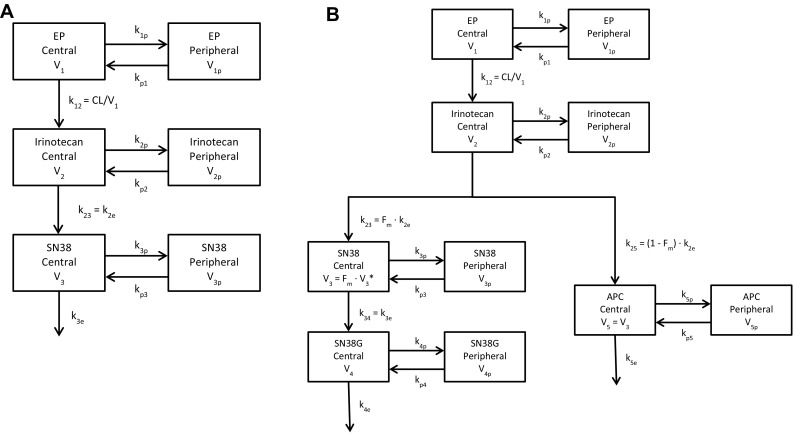
Model structure to describe etirinotecan pegol metabolism using a 3-analyte model (**a**) and 5-analyte model (**b**)

Prior to the development of an integrated pharmacokinetic model, two- and three-compartment pharmacokinetic models were evaluated for each analyte using data from study 06-IN-IR001 (see supplemental material). Fitting a three-compartment model to EP or any of its metabolites was either not successful or resulted in a non-parsimonious fit with greater variability in parameter estimates as compared to fitting with a two-compartment model. Hence, we decided to use a two-compartment model for each analyte in the integrated model. A graphical representation of the 3-analyte model is shown in Fig. 2a. As EP is administered by intravenous infusion, dosing starts in the first compartment, the central compartment for EP. Irinotecan is subsequently released from the four-arm polymer backbone. The transformation rate $\frac{{{F_{12}}{\text{CL}}}}{{{V_1}}}$ for the release of irinotecan represents the formation of irinotecan regardless of whether the original precursor has one, two, three, or four irinotecan moieties still attached; this rate constant, hence, is the aggregate release rate describing irinotecan formation. Irinotecan is subsequently metabolized to either SN38 or APC. In the 3-analyte model, this rate is represented by ${F_{23}}{k_{2{\text{e}}}}$. $V_{3}^{*}$ is the aggregated single term for ${V_3}/({F_{12}} \cdot {F_{23}})$. Hence, estimated volumes of distribution are not the true volumes of the analytes in humans, with the exception of EP volume of distribution. The scaling factor for the central and peripheral compartments for each analyte is represented by ${V_i}$ and ${V_{i{\text{p}}}}$, respectively, where *i* assumes numerical values starting with 1, representing each analyte. The elimination rate constants are ${k_{i0}}$, with increasing numerical values for *i* representing each analyte. The rate constants ${k_{i{\text{p}}}}$ and ${k_{{\text{p}}i}}$ represent the transfer from the central to the peripheral compartments and vice versa, respectively. A linear elimination process for all analytes was assumed, resulting in the following equations:

2 $$\frac{{{\text{d[EP]}}}}{{{\text{d}}t}}=\frac{{{\text{Dose}}}}{{\left( {{\text{Infusion}}\,{\text{Duration}}} \right) \cdot {V_1}}} - \left( {\frac{{{\text{CL}}}}{{{V_1}}}+{k_{1{\text{p}}}}} \right)[{\text{EP}}]+{k_{{\text{p}}1}}[{\text{E}}{{\text{P}}_{\text{p}}}],$$

3 $$\frac{{{\text{d}}[{\text{Irinotecan}}]}}{{{\text{d}}t}}=\frac{{{\text{CL}}}}{{V_{2}^{*}}}[{\text{EP}}] - ({k_{2{\text{e}}}}+{k_{2{\text{p}}}}) \cdot [{\text{Irinotecan}}]+{k_{{\text{p}}2}}[{\text{Irinoteca}}{{\text{n}}_{\text{p}}}],$$

where $$V_{2}^{*}={V_2}/{F_{12}}$$

4 $$\frac{{{\text{d}}[{\text{SN}}38]}}{{{\text{d}}t}}=\frac{1}{{V_{3}^{*}}} \cdot {k_{2{\text{e}}}} \cdot {V_2}[{\text{Irinotecan}}] - ({k_{{\text{3e}}}}+{k_{3{\text{p}}}}) \cdot [{\text{SN}}38]+{k_{{\text{p}}3}}[{\text{SN}}{38_{\text{p}}}],$$

where $V_{3}^{*}={V_3}/({F_{12}} \cdot {F_{23}})$ for the 3-analyte model.

${\text{CL}}/{V_1}$ represents both the EP elimination rate and its conversion to irinotecan; ${k_{{\text{2e}}}}$ is the rate constant for irinotecan elimination and its conversion to SN38; ${k_{3{\text{e}}}}$ for SN38 elimination and its conversion to SN38G. Metabolite conversion fractions and volumes of distribution are non-identifiable, and thus, only ratios of metabolic conversion fraction to volume of distribution were estimated. The *F* and *V* parameters were indistinguishable by the model, and thus, aggregate parameters represented by $V_{i}^{*}$ for $i=2,3$ were determined instead. For example, ${F_{12}}$ and ${V_{2~}}$ cannot be separately distinguished by the model, and thus, $V_{2}^{*}={V_2}/{F_{12}}$ was determined. The same approach was applied to the other volume terms.

**Fig. 3:**
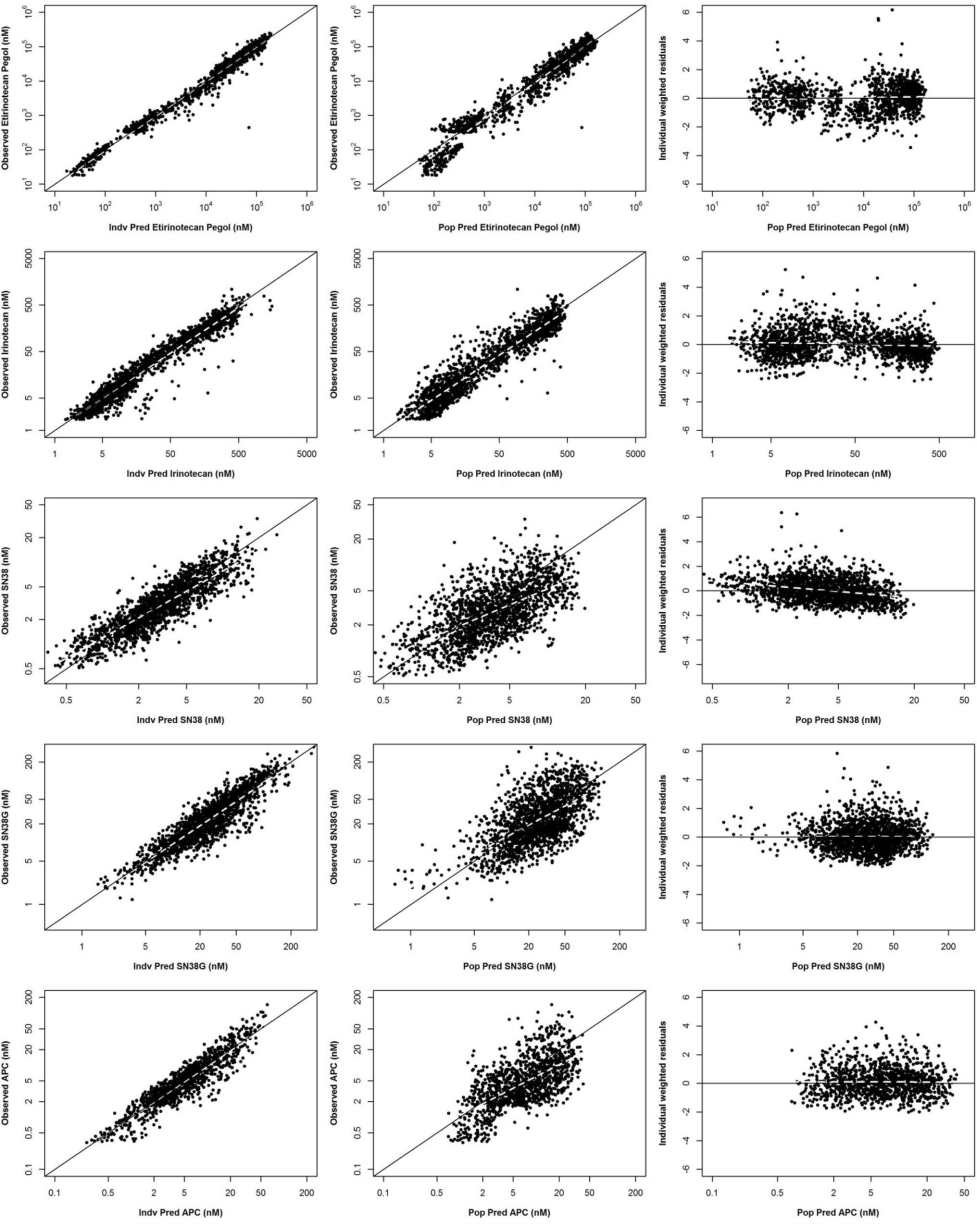
Goodness-of-fit plots for all analytes of etirinotecan pegol from the intital 3-analyte (**a**) and final 5-analyte (**b**) population pharmacokinetic models. Left, observed vs. individual-predicted concentrations; center, observed vs. population-predicted concentrations; right, individual weighted residuals vs. population-predicted concentrations. Solid lines represent the line of unity in left and center and zero residuals in right. Dashed lines represent loess smoothing

Goodness-of-fit plots for all analytes showed good agreement between individual-predicted and observed concentrations without noticeable bias in individual weighted residuals across predicted drug concentration values (first 3 rows of Fig. 3). The majority of individual weighted residuals were within the ± 2 units from the zero ordinate.

Graphical outputs for covariate screening are shown in Figures S2 to S5 in supplemental material. The full covariate model for the 3-analyte model was the first ranked model in Table 3, which included BSA on CL and *V*_1_, age on CL, and UGT1A1 on *k*_3e_; eGFR was a significant covariate of CL in subsequent stepwise addition. The parameters identified as impacted by covariates are described below:

**Table 3:** Top 10 covariate models based on the WAM algorithm, ranked by Schwartz Bayesian criterion

Rank	Covariate parameter	Approximated − 2 log likelihood (Λ′)	SBC′
1	${\theta _{{\text{CL,AGE}}}},~{\theta _{{\text{CL,BSA}}}},~{\theta _{{V_1},{\text{BSA}}}},{\theta _{{k_{3{\text{e}}}},{\text{~UGT1A1}}}}$	13.4	− 40.72
2	${\theta _{{\text{CL,AGE}}}},~{\theta _{{\text{CL,BSA}}}},~{\theta _{{\text{CL,eGFR}}}},~{\theta _{{V_1},{\text{BSA}}}},~{\theta _{{k_{3{\text{e}}}},~{\text{UGT1A1}}}}$	5.6	− 41.08
3	${\theta _{{\text{CL,AGE}}}},~{\theta _{{\text{CL,BSA}}}},~{\theta _{{V_1},{\text{BSA}}}},{\theta _{{k_{3e}},~{\text{SEX}}}},{\theta _{{k_{3{\text{e}}}},~{\text{UGT1A1}}}}~$	8.2	− 42.38
4	${\theta _{{\text{CL,AGE}}}},~{\theta _{{\text{CL,BSA}}}},~{\theta _{{V_1},{\text{BSA}}}}$	25.3	− 42.41
5	${\theta _{{\text{CL,BSA}}}},{\theta _{{\text{CL,eGFR}}}},{\theta _{{V_1},{\text{BSA}}}},~{\theta _{{k_{3{\text{e}}}},~{\text{UGT1A1}}}}$	17.0	− 42.51
6	${\theta _{{\text{CL,AGE}}}},~{\theta _{{\text{CL,BSA}}}},~{\theta _{{\text{CL,eGFR}}}},~{\theta _{{V_1},{\text{BSA}}}},~{\theta _{{k_{3{\text{e}}}},~{\text{SEX}}}},{\theta _{{k_{3{\text{e}}}},~{\text{UGT1A1}}}}$	0.4	− 42.75
7	${\theta _{{\text{CL,AGE}}}},~{\theta _{{\text{CL,BSA}}}},~{\theta _{{\text{CL,eGFR}}}},~{\theta _{{V_1},{\text{BSA}}}}$	17.5	− 42.79
8	${\theta _{{\text{CL,AGE}}}},{\theta _{{\text{CL,BSA}}}}~,~{\theta _{{V_1},{\text{BSA}}}},{\theta _{{k_{3{\text{e}}}},~{\text{SEX}}}}$	17.9	− 43.00
9	${\theta _{{\text{CL,AGE}}}},~{\theta _{{\text{CL,BSA}}}},{\theta _{{\text{CL,eGFR}}}},{\theta _{{V_1},{\text{BSA}}}},{\theta _{{k_{3{\text{e}}}},~{\text{SEX}}}}$	10.2	− 43.38
10	${\theta _{{\text{CL,BSA}}}},{\theta _{{\text{CL,eGFR}}}},~{\theta _{{V_1},{\text{BSA}}}}~$	28.6	− 44.10

*SBC* Schwartz Bayesian criterion

5 $${\text{EP}}\,{\text{CL}}={\theta _{{\text{CL}}}} \cdot {\left( {\frac{{{\text{AGE}}}}{{60}}} \right)^{{\theta _{{\text{CL,AGE}}}}}} \cdot {\left( {\frac{{{\text{BSA}}}}{{1.86}}} \right)^{{\theta _{{\text{CL,BSA}}}}}} \cdot {\left( {\frac{{{\text{eGFR}}}}{{84.1}}} \right)^{{\theta _{{\text{CL,eGFR}}}}}}\exp ({\eta _{{\text{CL}}}})$$

6 $${V_1}={\theta _{{V_1}}} \cdot {\left( {\frac{{{\text{BSA}}}}{{1.86}}} \right)^{{\theta _{{V_1},{\text{BSA}}}}}}\exp ({\eta _{{V_1}}})$$

7 $${k_{3{\text{e}}}}={\theta _{{k_{3{\text{e}}}}}}\exp \left( {{\theta _{{k_{3{\text{e}}}},{\text{UGT1A1}}}} \cdot I\left[ {{\text{UGT1A1}}={\text{TA}}(7)/{\text{TA}}(7)} \right]} \right) \cdot \exp \left( {{\eta _{{k_{3{\text{e}}}}}}} \right).$$

Table 4 provides a summary of the parameter estimates for the 3-analyte model. The aggregate effect of incorporating covariates to EP CL and *V*_1_ parameters reduced interindividual variability expressed in CV% from 34 to 27% and from 26 to 22%, respectively. UGT1A1 polymorphism as a covariate of *k*_3e_ reduced the interindividual variability of *k*_3e_ from 51 to 47%. The magnitude of the covariate effects was small.

**Table 4:** Summary of final parameter estimates in the 3-analyte (EP, Irinotecan, SN38) in a typical patient population consisting of age = 60 years, BSA = 1.86 m^2^, eGFR = 79.1 mL/min, and heterozygous UGT1A1*28 genotype

Analyte	Parameter	Population parameter, estimate ± SE	Interindividual variability, variance ± SE (CV%)
*3-analyte model*			
EP	CL (L/h)	0.237 ± 0.008	0.07654 ± 0.014 (27%)
	*V*_1_ (L)	5.05 ± 0.14	0.051 ± 0.01 (22%)
	*k*_1p_ (h^−1^)	6.78 × 10^−3^ ± 5.5 × 10^−4^	0.295 ± 0.07 (54%)
	*k*_p1_ (h^−1^)	5.8 × 10^−4^ ± 4.1 × 10^−5^	**–**
	${\theta _{{\text{CL,AGE}}}}$	− 0.271 ± 0.084	–
	${\theta _{{\text{CL,BSA}}}}$	1.32 ± 0.23	–
	${\theta _{{\text{CL,eGFR}}}}$	0.2 ± 0.068	–
	${\theta _{{V_1},{\text{BSA}}}}$	1.1 ± 0.19	–
	Corr (CL, *V*_1_)	0.759 ± 0.058	–
	Multiplicative error	0.289 ± 0.0072	–
Irinotecan	$V_{2}^{*}={V_2}/{F_{12}}$ (L)	1.8 ± 0.13	0.354 ± 0.062 (59%)
	*k*_2e_ (h^−1^)	27.6 ± 1.7	0.204 ± 0.045 (45%)
	*k*_2p_ (h^−1^)	18.8 ± 1.2	0.214 ± 0.04 (46%)
	*k*_p2_ (h^−1^)	3.2 × 10^−3^ ± 1.5 × 10^−4^	–
	Corr (*k*_2e_, $V_{2}^{*}$)		− 0.751 ± 0.064
	Multiplicative error	0.376 ± 0.0081	–
SN38	$$V_{3}^{{**}}=\frac{{{V_3}}}{{{F_{12}}{F_{23}}}}({\text{L}})$$	80 ± 190	0.14 ± 0.028 (37%)
	*k*_3e_ (h^−1^)	0.0602 ± 0.0042	0.224 ± 0.048 (47%)
	*k*_3p_ (h^−1^)	0.23 ± 0.023	0.561 ± 0.12 (75%)
	*k*_p3_ (h^−1^)	8.75 × 10^−3^ ± 5.4 × 10^−4^	–
	${\theta _{{k_{3{\text{e}}}},{\text{UGT1A1}}}}$	− 0.67 ± 0.21	–
	Multiplicative error	0.34 ± 0.0089	–
	Additive error	0.383 ± 0.033	–
*5-analyte model*			
SN38G	$V_{4}^{*}=\frac{{{V_4}}}{{{F_{12}}({F_{23}}+{F_{25}}){F_{34}}}}({\text{L}})$	11.6 ± 0.95	0.415 ± 0.081 (64%)
	*k*_4e_ (h^−1^)	1.41 ± 0.093	0.232 ± 0.043 (48%)
	*k*_4p_ (h^−1^)	0.548 ± 0.056	–
	*k*_p4_ (h^−1^)	0.104 ± 0.014	–
	Multiplicative error	0.392 ± 0.0083	–
APC	(*F*_irinotecan→SN38_)	0.631 ± 0.017	0.0528 ± 0.012 (23%)
	*k*_5e_ (h^−1^)	0.0235 ± 0.0027	0.479 ± 0.13 (69%)
	*k*_5p_ (h^−1^)	0.0236 ± 0.003	0.664 ± 0.2 (81%)
	*k*_p5_ (h^−1^)	0.00139 ± 0.00018	–
	Multiplicative error	0.356 ± 0.0092	–
Correlations	Corr (CL, *V*_1_)	–	0.713 ± 0.056
	Corr (*k*_2e_, *V*_2_)	–	− 0.755 ± 0.046

#### 5-Analyte pharmacokinetic model

Upon completion of the final 3-analyte model, individual parameter estimates for EP, irinotecan, and SN38 were fixed [with the exception for the Corr (${\text{CL}},{V_1}$) and Corr (${k_{2{\text{e}}}},V_{2}^{*}$) which could not be fixed in the software], and SN38G and APC concentration–time data were added to the model, thus generating the 5-analyte model, graphically represented in the last two rows in Fig. 3. Assuming linear elimination processes for SN38G and APC, the following additional equations were incorporated:

8 $$\frac{{{\text{d}}[{\text{SN}}38{\text{G}}]}}{{{\text{d}}t}}=\frac{1}{{V_{4}^{*}}} \cdot {k_{4{\text{e}}}} \cdot {V_3}[{\text{SN}}38] - ({k_{4{\text{e}}}}+{k_{4{\text{p}}}}) \cdot \left[ {{\text{SN}}38{\text{G}}} \right]+{k_{{\text{p}}4}}[{\text{SN}}38{{\text{G}}_{\text{p}}}],$$

where $$V_{4}^{*}={V_4}/({F_{12}} \cdot ({F_{23}}+{F_{25}}) \cdot {F_{34}})$$

9 $$\frac{{{\text{d}}[{\text{APC}}]}}{{{\text{d}}t}}=\frac{1}{{V_{5}^{*}}} \cdot {k_{2{\text{e}}}} \cdot {V_2}[{\text{Irinotecan}}] - ({k_{5{\text{e}}}}+{k_{5{\text{p}}}}) \cdot [{\text{APC}}]+{k_{{\text{p}}5}}[{\text{AP}}{{\text{C}}_{\text{p}}}],$$

where $$V_{5}^{*}={V_5}/{F_{25}}=V_{3}^{*}$$.

$V_{3}^{*}$ in this 5-analyte model represents the aggregated term for ${V_3}/({F_{12}} \cdot ({F_{23}}+{F_{25}}))$. Glucuronidation of SN38 to SN38G is governed by the rate constant ${F_{34}}{k_{3{\text{e}}}}$. The volume of the central compartment for APC was assumed to be the same as that for SN38. The fraction for the formation of SN38 (*F*_irinotecan→SN38_) and subsequently one minus that fraction (1 − *F*_irinotecan→SN38_) to describe the formation of APC accounted for the difference in the concentrations of the two metabolites. Hence, estimated volumes of distribution are not the true volumes for APC and SN38G in humans. The scaling factor for the central and peripheral compartments, elimination rate constants, and rate constants from the central to the peripheral compartments and vice versa for SN38G and APC are represented equivalent to those described for the 3-analyte model. As described for the 3-analyte model, only ratios of metabolic conversion fraction to volume of distribution were estimated.

Final parameters are shown in Table 4. In addition to the covariates identified in the 3-analyte model, the impact of UGT1A1 status on the ratio of central volume of distribution of SN38G to its conversion fractions ($V_{4}^{*}$) was evaluated; however, the covariate was not found significant. CYP3A4/5 inducer effect was significant on *k*_25_, which is the conversion of irinotecan to APC. However, the direction of the effect indicates that CYP3A4 inducers reduced the conversion of irinotecan to APC, contrary to the expected effect of CYP3A4/5 inducers. Thus, this covariate was not included in the final model. The 5-analyte model maintained all of the covariates in the 3-analyte model.

Goodness-of-fit plots for all analytes showed good agreement between individual-predicted and observed concentrations without noticeable bias in individual weighted residuals across predicted drug concentration values (Fig. 3). The majority of individual weighted residuals were within the ± 2 units from the zero ordinate.

Prediction- and variability-corrected visual predictive checks, stratified by dosing schedules, in supplemental material Figures S6 and S7, captured the majority of the observed data within the 95% prediction interval.

### Clinical impact of significant covariates

**Fig. 4:**
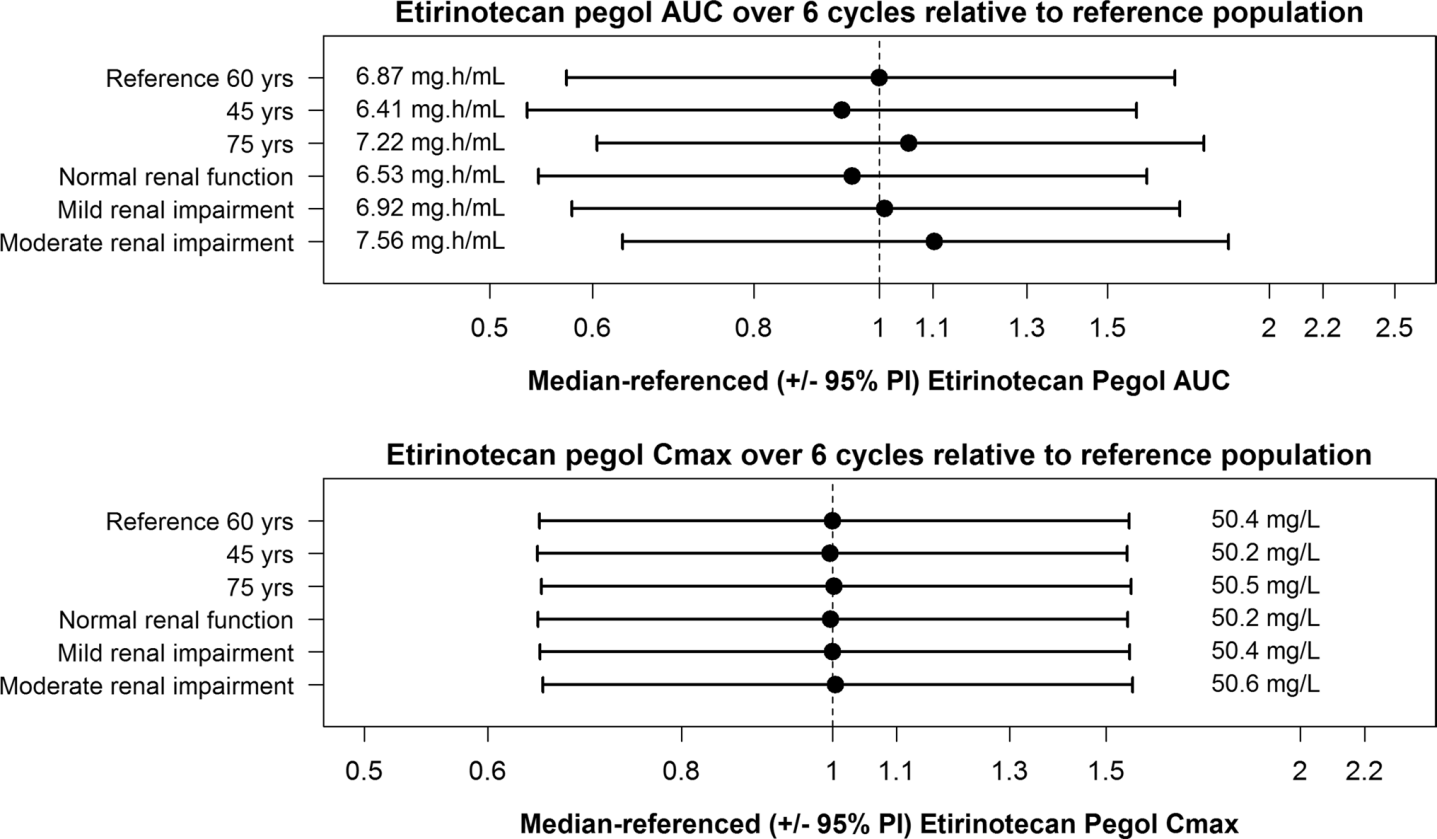
Forest plots showing the effect of age and eGFR on the cumulative AUC and *C*_max_ of etirinotecan pegol over six cycles relative to a reference population. The eGFR used in each category was the midpoint of the normal renal function and mild, moderate and severe renal impairment groups. The reference population reflects the typical patient’s characteristics, i.e., demographics, baseline lab values and UGT1A1 status, in phase I and II studies

To assess the clinical impact of significant covariates, we simulated exposure for specified patient populations. A reference population was generated by setting each covariate to the median values of those in the study population for age (60 years) and renal function (84.1 mL/min), and UGT1A1 to non-homozygous UGT1A1*28. To assess the clinical relevance of age, renal function, and UGT1A1*28, their impacts on EP and SN38 exposure were evaluated by simulating 500 virtual patients in the following scenarios: (1) 75 years old; (2) 45 years old; (3) 45 mL/min eGFR for moderate renal impairment; (4) 75 mL/min eGFR for mild renal impairment; (5) 105 mL/min eGFR for normal renal function. Figure 4 shows forest plots of the impact of age and eGFR on EP AUC and *C*_max_. In the top panel, the EP exposures in these scenarios were close to that of the reference. The 95% prediction interval of each scenario overlapped considerably, suggesting limited clinical impact. As illustrated in the bottom panel of Fig. 4, EP *C*_max_ was not impacted by any of the covariates.

**Fig. 5:**
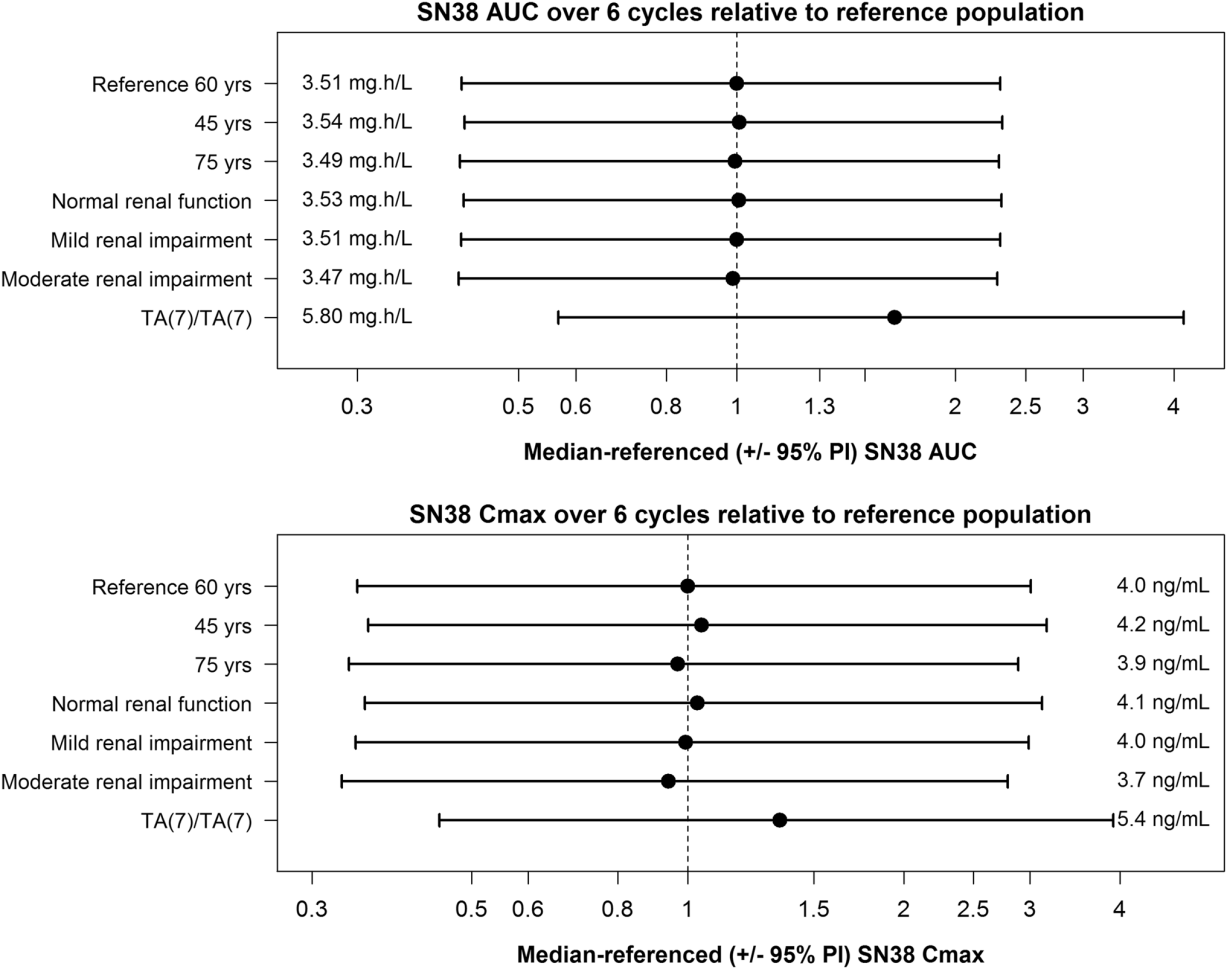
Forest plots showing the effect of UGT1A1 polymorphism, age, and eGFR on cumulative SN38 AUC and *C*_max_ values of over six treatment cycles relative to a reference population. The eGFR used in each category was the midpoint of the normal renal function and mild, moderate, and severe renal impairment groups. The reference population reflects the typical patient’s characteristics, i.e., demographics, baseline lab values, and UGT1A1 status, in phase I and II studies

In addition to assessing the impact of age and renal function on EP exposure, their impact on SN38 exposure was also investigated. Depicted in Fig. 5, and consistent with the limited impact of age and eGFR on EP exposure, the downstream effects on SN38 AUC and *C*_max_ were negligible. In comparison to a patient who is wild-type or heterozygous for the UGT1A1 promoter region polymorphism, the medians of the estimated SN38 AUC and *C*_max_ increased by about 1.7- and 1.3-fold, respectively, for a patient homozygous for UGT1A1*28. The evaluation of the impact of UGT1A1*28 on SN38 exposure was based on a limited sample size, as only nine patients in the data set were homozygous for UGT1A1*28 and additional data are required to adequately assess the true magnitude of the impact of UGT1A1*28 on SN38 pharmacokinetics and the potential clinical implication for EP safety.

## Discussion

The pharmacokinetics of EP and its metabolites irinotecan, SN38, SN38G, and APC were well described by a semi-mechanistic model that included all five analytes. The integrated model of all analytes describes known mechanisms of the EP metabolic pathway. Nonetheless, limitations to the model do exist. Due to the complexity of the model, the fractions that characterize a specific metabolic direction and the volume of distribution of the central compartment of the metabolite were aggregated into a single volume parameter. Even with these limitations, the integrated model was sufficient for characterization of the individual concentration–time profile for all analytes and enabled subsequent simulations of the effects of various characteristics of the population.

The clinical relevance of the observed covariate effects was evaluated through a series of simulations with varying covariate values. Baseline BSA had an effect on EP clearance and volume of distribution, supporting the BSA-based dosing of EP. An additional covariate for EP clearance was eGFR, consistent with its elimination pathways, hydrolysis to irinotecan, and renal clearance. EP clearance decreases with decreasing kidney function. However, the effect of mild and moderate renal impairment was minimal. The small increase in EP exposure with increasing renal impairment had no impact on the exposure of the active metabolite SN38. Consequently, dose adjustments are not required in patients with mildly or moderately impaired renal function.

Age was a significant covariate of EP CL. However, varying the age from 45 to 75 years had minimal impact on EP or SN38 exposures, suggesting that no dose adjustment of EP is required in the elderly population. In addition to the covariates impacting EP clearance, the UGT1A1*28 polymorphism was identified as a statistically significant covariate for SN38 elimination. The increase in the number of TA repeats in its promoter region is associated with decreased enzymatic activity [21, 22]. Patients homozygous for UGT1A1*28 genotype were projected to exhibit a 1.8-fold higher SN38 exposure, which could potentially impact the safety and tolerability of EP. However, the variability explained by UGT1A1 polymorphism was relatively small. The maximum SN38 concentrations resulting from EP administration are lower than those for a 350 mg/m^2^ as well as a 60 mg/m^2^ irinotecan single dose administration [8]; the standard dosing regimen for irinotecan is 350 mg/m^2^ once every 3 weeks in colon cancer patients. A toxicokinetic study in dogs comparing irinotecan and EP showed that SN38 *C*_max_ but not AUC was associated with drug-induced neutropenia [10]. A meta-analysis consisting of 878 patients reported that the association between UGT1A1*28 genotype and irinotecan-induced hematologic toxicity was significant only at higher irinotecan doses that resulted in higher maximum SN38 concentrations [23]. Another meta-analysis consisting of 1998 patients suggested that a dose-dependent association existed between homozygous UGT1A1*28 patients and the risk of irinotecan-induced neutropenia with the high dose (≥ 250 mg/m^2^) compared to the low (80–145 mg/m^2^) and medium (150–200 mg/m^2^) doses (relative risk: 7.22 vs. 2.04) [24]. A separate meta-analysis by the latter group also found a dose-dependent increased risk of diarrhea and UGT1A1*28 homozygotes at medium and high doses but not at low doses of irinotecan [25]. We recognize that the small number of homozygous UGT1A1*28 patients in the current analysis population limits broad generalization of the true impact of UGT1A1*28 polymorphism in the general population.

In conclusion, EP pharmacokinetics are well described by the proposed model. Other than the effect of BSA, which is already taken into account by a BSA-based dosing scheme, no other covariates were deemed to have clinical implications. No EP dose adjustment based on the covariates investigated appears necessary.

## Electronic supplementary material

Below is the link to the electronic supplementary material.

Supplementary material 1 (DOCX 467 KB)
