# Supplementary material for: Integrated population pharmacokinetics of etirinotecan pegol and its four metabolites in cancer patients with solid tumors
Source: Cancer Chemother Pharmacol. 2018 Mar 21;81(5):897–909. doi: 10.1007/s00280-018-3562-3 (PMC5907632; doi:10.1007/s00280-018-3562-3)
Supplement: Supplementary file 1 — Supplementary material 1 (DOCX 467 KB) [file 280_2018_3562_MOESM1_ESM.docx]

**Supplemental Materials**

**Exclusion of patients from analysis**

Eleven patients (9 from 06-IN-IR001; 2 from 07-PIR-02) were excluded from analysis due to the occurrence of unexpectedly high irinotecan concentrations, ranging from 2- to over 1000-fold difference. The prevention of EP hydrolysis to irinotecan was dependent on proper handling conditions during blood collection and the plasma harvesting process. Since the abnormally high irinotecan concentration levels were only observed from patients treated at specific sites, improper sample handling was suspected and patient’s profiles were removed from analysis. The observed high irinotecan concentration had no impact on patient safety.

**2- vs. 3-compartment structural models for etirinotecan pegol (EP) and its metabolites**

Both 2- and 3-compartmental models using sum-of-exponential equations within the nonlinear mixed effects modeling framework in Monolix 3.1 were fitted to the concentration-time profiles of each of the 5 analytes of EP in patients who received EP at a dosing schedule of wx3 q4wk, q14d, and q21d from study 06-IN-IR001. Comparison of modelling results for 2- and 3-compartment model is shown in Table S1. Overall, fitting a 3-compartment model to etirinotecan pegol or any of its metabolites was either not successful or resulted in a non-parsimonious fit with greater variability in parameter estimates as compared to fitting with a 2-compartment model, thus validating our original choice.

**Table S1: Comparison of modeling results using 2- and 3-compartment models**

| **Drug or metabolite** | **2-Compartment**  **Model Results** | **3-Compartment**  **Model Results** | **Conclusions** |
| --- | --- | --- | --- |
| Etirinotecan Pegol | - Model converged | - Model converged - No overall improvement in fit; SEs of fixed and random effects greater than with 2-comp model - Plot of 3- vs. 2-comp IPRED values is linear with minimal variability (Figure S1) | - 2-comp model predicts NKTR-102 exposure equally well as a 3-comp model. - 3-comp model is overspecified for dataset - 2-comp model fits to data well and is parsimonious |
| Irinotecan | - Model converged | - Model did not converge; Monolix warning “Parameter Gamma cannot be correctly estimated”* issued throughout the fitting step. | - 2-comp model fits to data well and is parsimonious - 3-comp model is overspecified for dataset |
| SN38 | - Model converged | - Model did not converge; Monolix warning “Parameter Gamma cannot be correctly estimated”* issued throughout the fitting step. | - 2-comp model fits to data well and is parsimonious - 3-comp model is overspecified for dataset |
| SN38 Glucuronide | - Model converged | - Model did not converge; Monolix warning “Parameter Gamma cannot be correctly estimated”* issued throughout the fitting step. | - 2-comp model fits to data well and is parsimonious - 3-comp model is overspecified for dataset |
| APC | - Model converged | - Model did not converge; Monolix warning “Parameter Gamma cannot be correctly estimated”* issued throughout the fitting step. | - 2-comp model fits to data well and is parsimonious - 3-comp model is overspecified for dataset |

*Monolix warning “Parameter [name] cannot be correctly estimated” occurs “when a parameter does not influence the structural model”. Communication from H. Mesa, Monolix Software Development Engineer, Lixoft Forum, 25 March 2015

**Figure S1: Scatterplot of** **EP 3-compartment individual model predicted data (IPRED) and EP 2-compartment IPRED of data from Study 06-IN-IR001, N = 1357 observations**


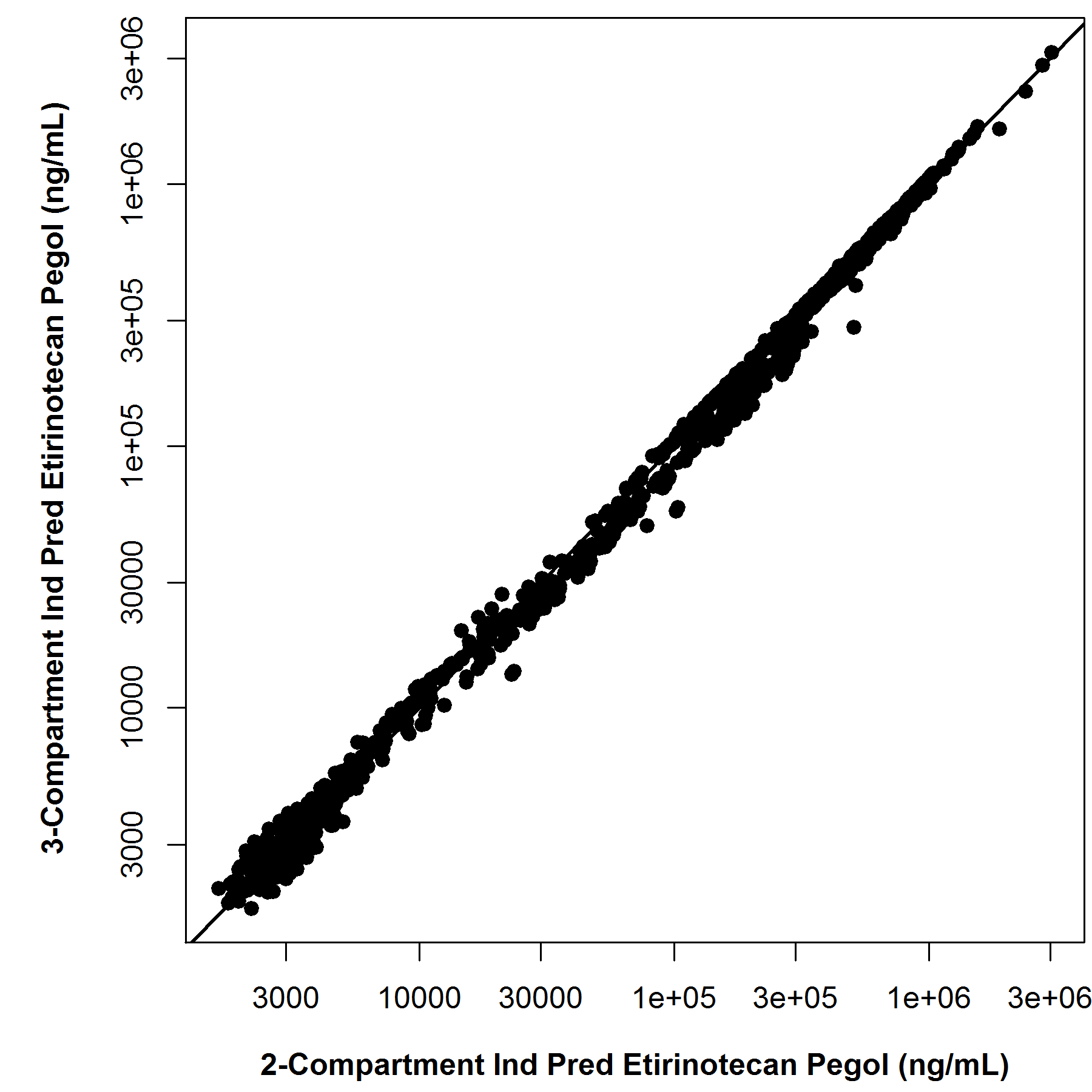


**Covariate selection**

Since the correlation between BSA and BW was 0.96, only BSA was further tested for significance. EP (NKTR-102) clearance appeared to increase with higher BSA (Figure S2), and in males (Figure S3); volume of distribution appeared to be lower with higher BSA (Figure S2). Males tended to have higher BSA compared to females. Thus, gender effect is likely confounded in BSA effect. In addition to BSA and gender, EP clearance appeared to be higher with increasing estimated glomerular filtration rate (Figure S4). For irinotecan, there was no noticeable trend in the covariates investigated. For SN38, visual inspection identified gender (males tended to have higher SN38 elimination) and UGT1A1*28 homozygosity (patients homozygous for UGT1A1*28 allele tended to have lower SN38 elimination) as potentially important covariates (Figure S5).

Among the tested covariates, the highest ranked model based on Wald approximation to likelihood ratio test in conjunction with Schwarz’s Bayesion criterion (SBC) maintained AGE and BSA as potentially significant covariates for EP clearance (*CL*), BSA as potentially significant for EP central volume (*V*_1_)*_,_* and UGT1A1 status as potentially important for *k*_3e_ (SN38 elimination). The potentially important effects of BSA on *CL*, and *V*_1_ were consistently found in the top models. Out of the top 10 models enumerated, AGE on CL appeared 8 times, while baseline estimated GFR on CL and UGT1A1 status and gender on *k_3e_* appeared 4-6 times as potential important covariates.

**Figure S2: Scatterplot of etirinotecan pegol (NKTR-102) clearance and volume of distribution versus baseline body surface area (top) and irinotecan and SN38 elimination rate constant versus BSA (bottom)**

**Figure S3: Etirinotecan pegol (NKTR-102), irinotecan, and SN38 pharmacokinetic parameters by gender**

**Figure S4: Scatterplot of etirinotecan pegol (NKTR-102) clearance and volume of distribution versus baseline serum creatinine (top) and versus estimated glomerular filtration rate at baseline (bottom)**

**Figure S5: SN38 elimination by gender (left) and by UGT1A1*28 allele copies (right)**

**Model qualification by visual predictive check**

Shown in Figure S6 stratified by dosing schedule, the prediction- and variability-corrected visual predictive check plots captured majority of the observed data within the 90% prediction intervals. The VPC were generated from 2000 simulations. The stratification of the VPC was based on dosing schedule, rather than covariates, in order to show cleaner trends in the plots. To further assess model fit for the schedule and dose intended for commercial use (q21d), we also compared the 90% prediction interval of the observed data (empirical) with that of the model-predicted data (theoretical) for patients that received etirinotecan pegol on the q21d schedule (Figure S7). There was a good overlap and agreement between the empirical and theoretical prediction intervals for all analytes, indicating that variability was characterized sufficiently in the final population PK model.

Figure S6: Prediction- and Variability-Corrected Visual Predictive Checks By Dosing Schedule

| **Q21d Schedule** | **Q14d Schedule** | **Wx3 q4wk Schedule** |
| --- | --- | --- |
| **Etirinotecan Pegol** |  |  |
| 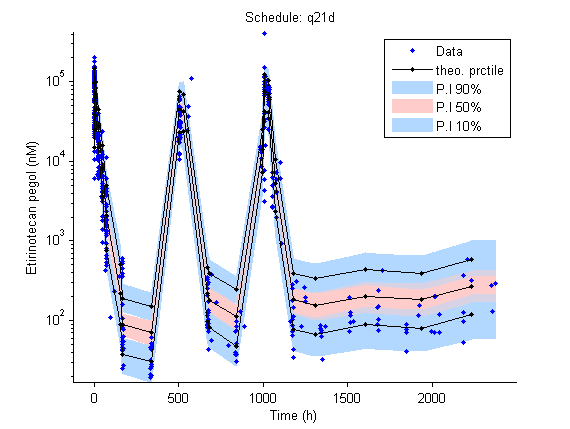 | 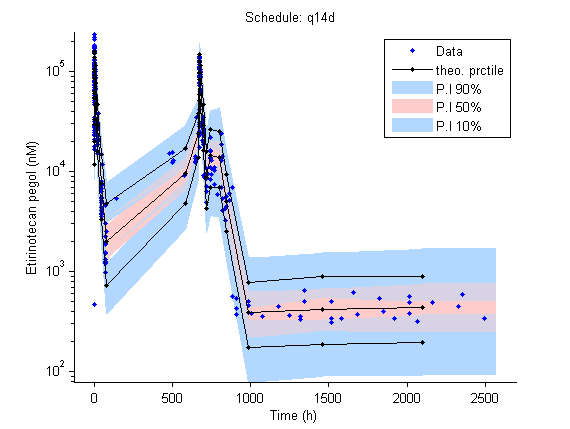 | 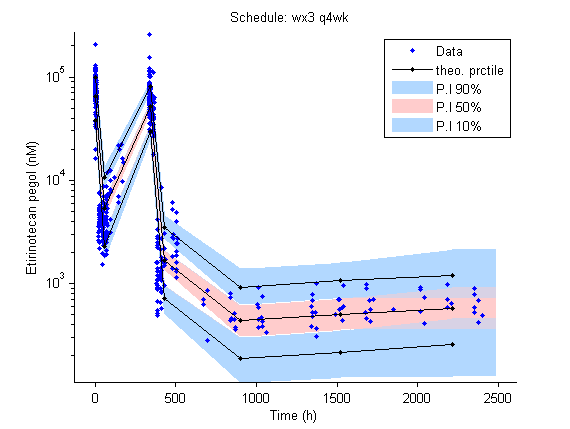 |
|  |  |  |
| **Irinotecan** |  |  |
| 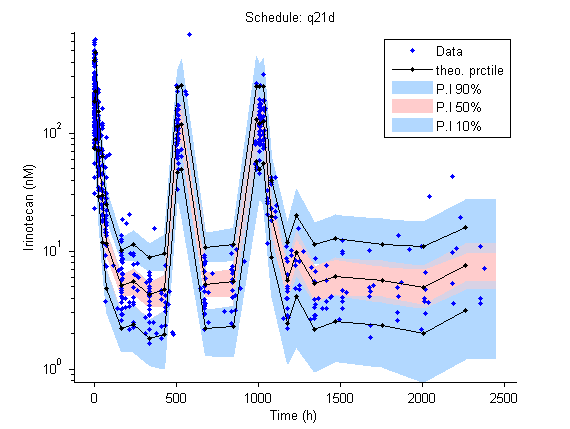 | 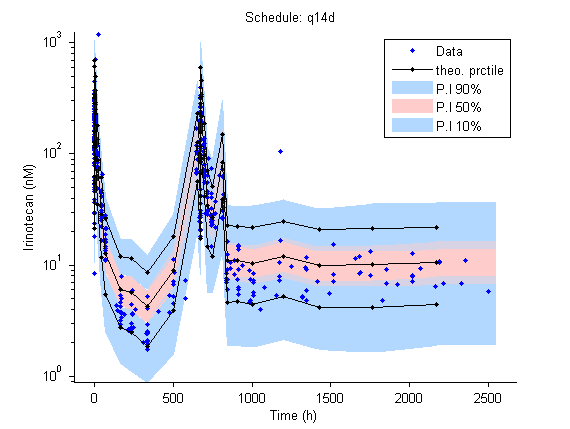 | 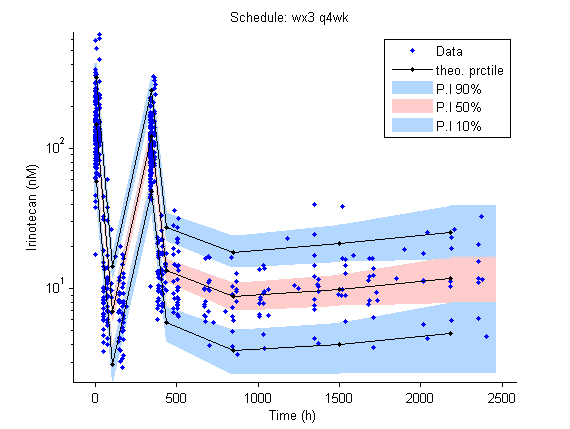 |
|  |  |  |
| **SN38** |  |  |
| 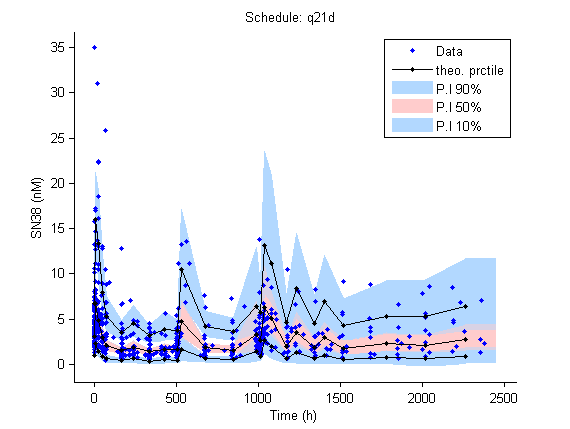 | 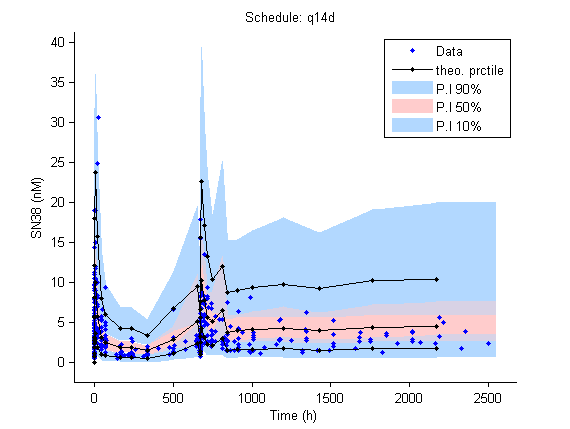 | 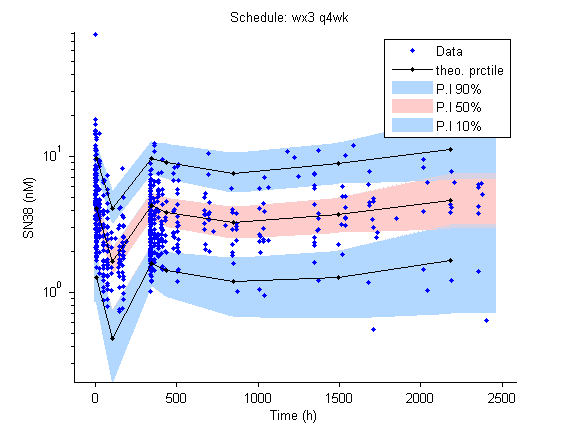 |
|  |  |  |
| **SN38G** |  |  |
| 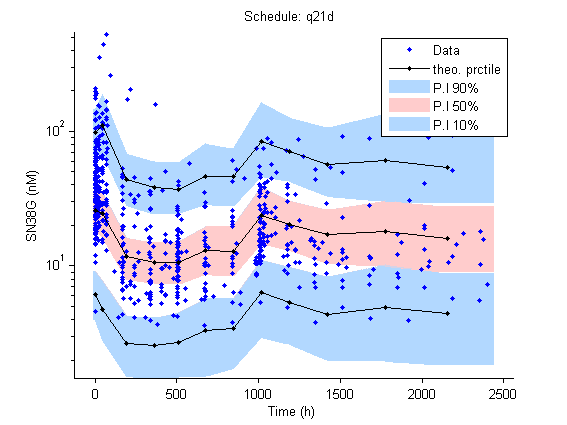 | 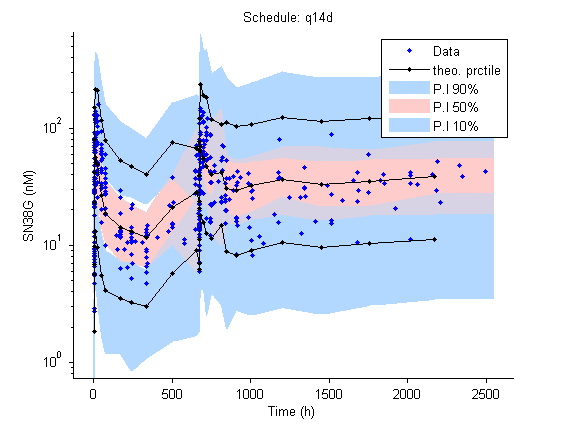 | 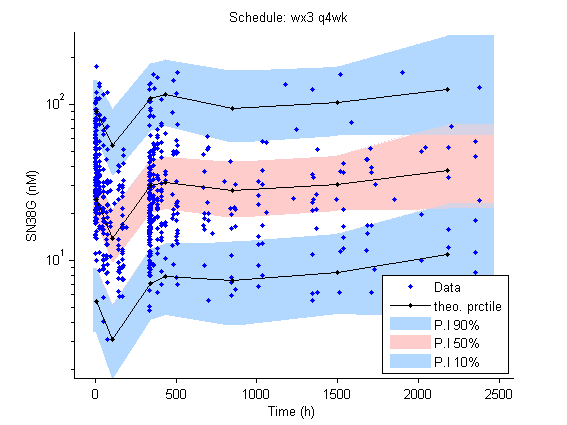 |
|  |  |  |
| **APC** |  |  |
| 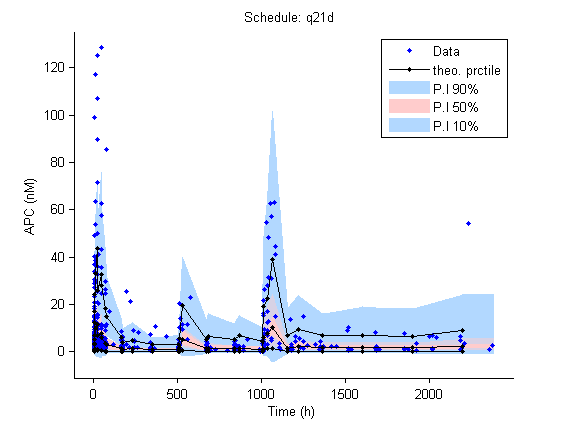 | 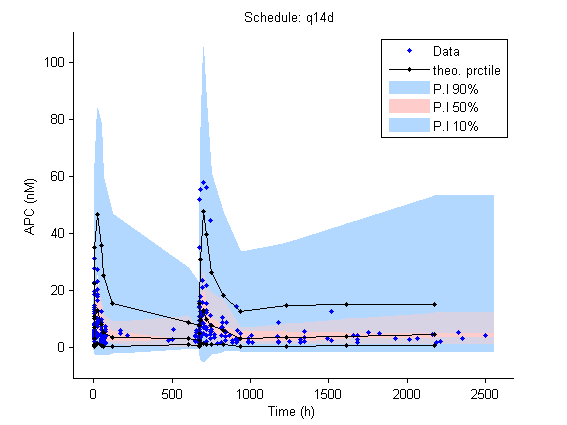 | 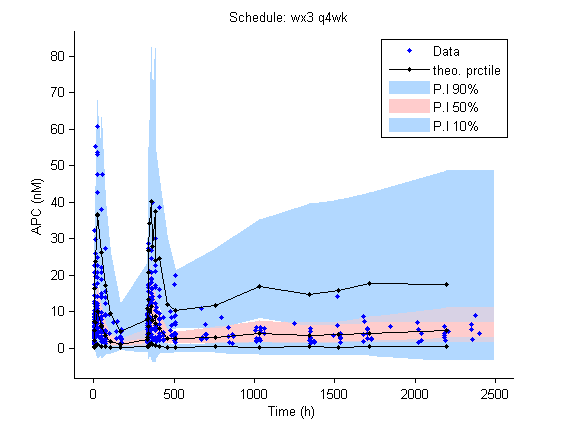 |
|  |  |  |

Circles represent measured data. Solid line indicates the theoretical percentile. Blue shading indicates 90% and 10% prediction interval. Orange shading represents the 50% prediction interval.

**Figure S7: Comparison of 95% prediction interval of observed (empirical) and model predicted (theoretical) data for patients receiving etirinotecan pegol q21d**

| **Etirinotecan Pegol** | **Irinotecan** | **SN38** |
| --- | --- | --- |
| 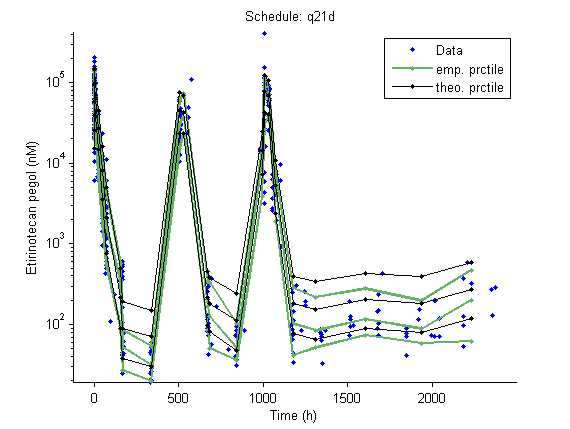 | 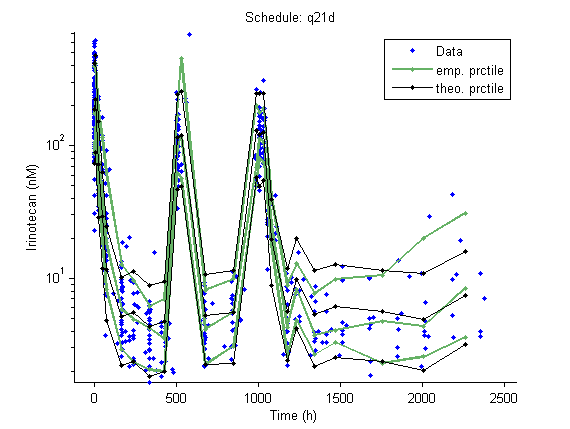 | 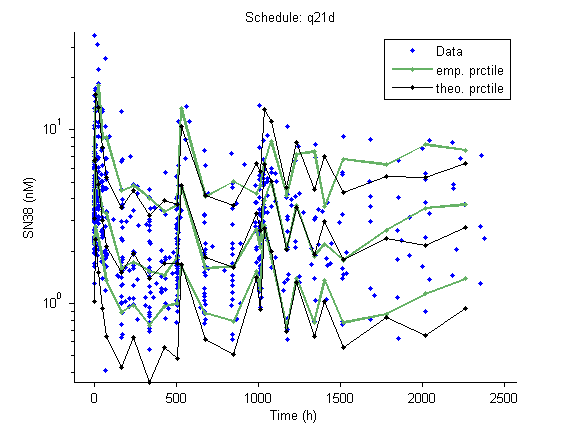 |
| **SN38G** | **APC** |  |
| 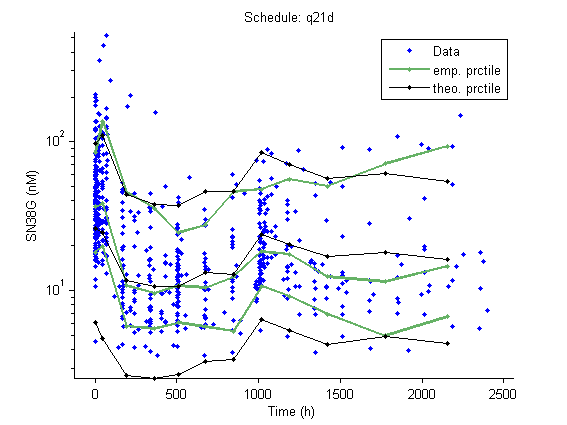 | 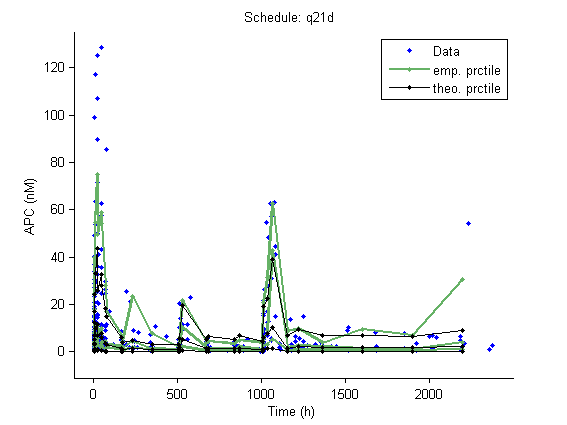 |  |
